# Supplementary material for: Friction modulation in limbless, three-dimensional gaits and heterogeneous terrains
Source: Nat Commun. 2021 Oct 19;12:6076. doi: 10.1038/s41467-021-26276-x (PMC8526626; doi:10.1038/s41467-021-26276-x)
Supplement: Supplementary file 1 — Supplementary Information [file 41467_2021_26276_MOESM1_ESM.pdf]

# – Supplementary Information –
Friction modulation in limbless, three-dimensional gaits and heterogeneous terrains

Xiaotian Zhang[1], Noel Naughton[1,2], Tejaswin Parthasarathy[1], and Mattia Gazzola[1,3,4,5]

[1]Department of Mechanical Science and Engineering,
[2]Beckman Institute for Advanced Science and Technology,
[3]National Center for Supercomputing Applications,
[4]Carl R. Woese Institute for Genomic Biology
[5]Center for Artificial Intelligence Innovation, University of Illinois at Urbana-Champaign

Correspondence and requests for materials should be addressed to M.G. (email: mgazzola@illinois.edu)

Contents

1 Snake trajectory as a particle in planar motion **2**

2 Definition of backward trajectories **3**

3 Steering rate, pose angle, and effective speed maps **4**
3.1 Impact of transverse friction ratio 4
3.2 Impact of Froude number 5
3.3 Impact of lateral to horizontal wavenumber ratio 5
3.4 Impact of modulation functional form 6

4 Simulating snakes using Cosserat rods **7**
4.1 Muscular activations and surrounding physics 7
4.2 Extended simulation methods and results 8
4.3 Simulation Parameters 10

Supplementary Note 1 — Snake trajectory as a particle in planar motion

In this note we provide the necessary derivations and justifications for analyzing the trajectory of the snake's center of mass as a particle on a planar trajectory analyzed using a polar coordinate frame. In the main text, the steering rate is denoted as $\dot{\theta}$, however, for notational convenience, in this note the steering rate will be denoted as $\dot{\Theta}$ in order to allow $\dot{\theta} = d\theta/dt$ in the usual manner for polar coordinates. The trajectory of the snake's center of mass $\bar{\mathbf{x}}(t)$ can be represented in polar coordinates $\bar{\mathbf{x}}(t) = (r\cos\theta, r\sin\theta)$ for an arbitrarily located origin point with unit vectors $\mathbf{u}_r = (\cos(\theta), \sin(\theta))$ and $\mathbf{u}_\theta = (-\sin(\theta), \cos(\theta))$. The velocity and acceleration of the particle can then be written as $\mathbf{v}(t) = \dot{r}\mathbf{u}_r + r\dot{\theta}\mathbf{u}_\theta$ and $\mathbf{a}(t) = (\ddot{r} - r\dot{\theta}^2)\mathbf{u}_r + (r\ddot{\theta} + 2\dot{r}\dot{\theta})\mathbf{u}_\theta$, respectively. Further, the norm of the particle's velocity is $|\mathbf{v}|^2 = \dot{r}^2 + (r\dot{\theta})^2$.

Substituting $r\dot{\theta}^2 = |\mathbf{v}|^2/r - \dot{r}^2/r$ into $\mathbf{a}(t)$ yields

$$\mathbf{a}(t) = \left(\ddot{r} - \frac{|\mathbf{v}|^2}{r} + \frac{\dot{r}^2}{r}\right)\mathbf{u}_r + \left(r\ddot{\theta} + 2\dot{r}\dot{\theta}\right)\mathbf{u}_\theta, \tag{1}$$

which can then be rearranged, multiplied by r and integrated over one undulation period to yield

$$\int_{t_0}^{t_1} r\mathbf{a}(t)\,dt = \int_{t_0}^{t_1}\left(-|\mathbf{v}|^2 + \frac{d}{dt}(\dot{r}r)\right)\mathbf{u}_r\,dt + \int_{t_0}^{t_1}\frac{d}{dt}\left(r^2\dot{\theta}\right)\mathbf{u}_\theta\,dt. \tag{2}$$

Finally, expanding out terms gives

$$\int_{t_0}^{t_1} r\mathbf{a}(t)\,dt = \left[-\int_{t_0}^{t_1}|\mathbf{v}|^2\,dt + \dot{r}(t_1)r(t_1) - \dot{r}(t_0)r(t_0)\right]\mathbf{u}_r + \left[r(t_1)^2\dot{\theta}(t_1) - r(t_0)^2\dot{\theta}(t_0)\right]\mathbf{u}_\theta. \tag{3}$$

The forcing functions $\kappa(s,t)$ and $\hat{N}(s,t)$ are periodic over the undulation interval $[t_0, t_1]$ and so it is reasonable to ask if the trajectory of the snake also exhibits periodicity over this same interval. If true, the time-averaged trajectory of the snake will necessarily be a circle (minus some inter-undulation period oscillations—see black lines in Fig. 2b) with the snake traversing some fraction of this circular trajectory every undulation period. Such a reduction would mean that the snake's behavior could be fully quantified based only on its trajectory over a single undulation period ($\mathcal{T} = \int_{t_0}^{t_1} dt$), greatly simplifying our analysis of the snake's behavior.

The assumption that the snake's overall trajectory is periodic and follows a circle requires that no inertial effects perturb the snake over an interval other than the undulation interval $[t_0, t_1]$ (i.e. there are no higher-order harmonic effects). This implies that the forcing functions on the particle trajectory must also be periodic over the undulation time interval $[t_0, t_1]$. This is true for integer values of the lateral to horizontal wave number ratio λ, which is always satisfied in our case.

Let us assume for the moment that such inertial effects do not occur. Under this assumption the time-averaged trajectory of the snake is indeed a circle, and one can define a new coordinate system with an origin point at its center. In this new coordinate systems, $\dot{r}(t_1)r(t_1) = \dot{r}(t_0)r(t_0)$ and $r(t_1)^2\dot{\theta}(t_1) = r(t_0)^2\dot{\theta}(t_0)$. These terms will then cancel in Eq. 3 yielding (after taking the L2 norm)

$$\left|\int_{t_0}^{t_1} r(t)\mathbf{a}(t)\,dt\right| = \int_{t_0}^{t_1}|\mathbf{v}(t)|^2\,dt. \tag{4}$$

Combining Eq. 4 with $Fr\,\bar{\mathbf{x}}_{tt}(t) = \mathbf{C}(t)$ from the Methods section (where $\mathbf{C}(t) = \int_0^1 -N(s,t)\boldsymbol{\mu}(s,t)\,ds$) allows us to write

$$\left|\int_{t_0}^{t_1} r(t)\,\mathbf{C}(t)\,dt\right| = Fr\int_{t_0}^{t_1}|\mathbf{v}(t)|^2\,dt. \tag{5}$$

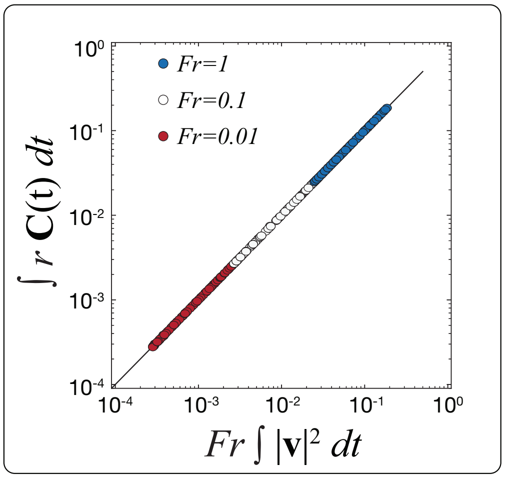

Supplementary Figure 1. Numerically evaluated LHS and RHS of Eq. 5 are computed at each simulation timestep and summed over one undulation period. Agreement with Eq. 5 indicates that there are no non-periodic or higher-order inertial effects influencing the snake's trajectory and thus one undulation period sufficiently describes the steady state trajectory of the snake.

We empirically validate that Eq. 5 holds in the case of our snake model by plotting the LHS vs RHS in Supplementary Fig. 1 for $\lambda = 1$ and a range of Froude numbers, demonstrating a perfectly linear relationship. Computationally, the transformed origin was found by fitting a circle to the snake's trajectory over ten undulation periods (collected after the ten startup periods). Ten periods are used to provide robustness to small numerical errors in the trajectory. The origin associated with Eq. 5 then enables trajectories to be quantified using the simple metrics of effective velocity $\mathbf{v}_{\text{eff}} = \int_{t_0}^{t_1} r\dot{\theta}\,\mathbf{u}_\theta\,dt/\mathcal{T}$ and steering rate $\dot{\Theta} = \int_{t_0}^{t_1} \dot{\theta}\,dt/\mathcal{T}$. We conclude by noting that further analysis of Eq. 5 is impaired due to the highly non-linear nature of $\boldsymbol{\mu}(s,t)$, which has a complex dependence on the local velocity along the snake's body.

Supplementary Note 2 — Definition of backward trajectories

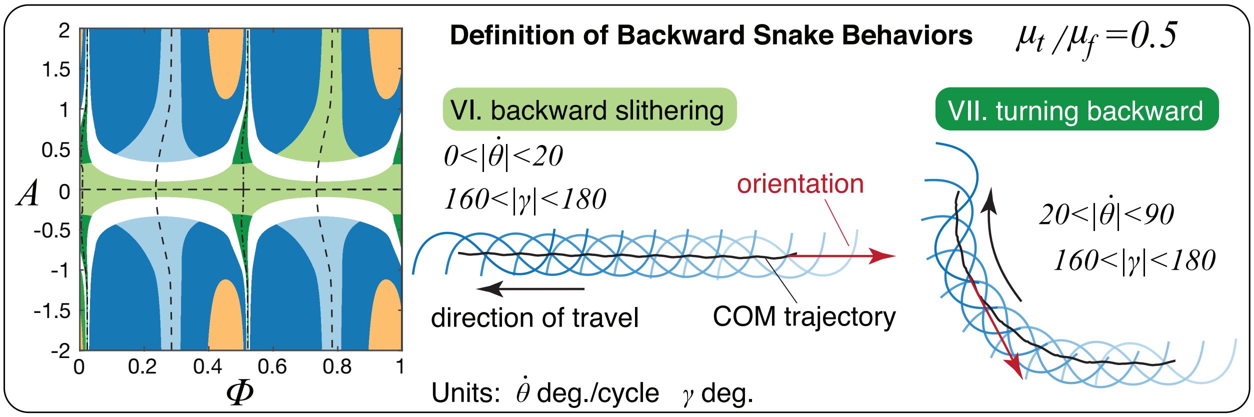

Supplementary Figure 2. Classification criteria for backwards locomotion behaviors found for $\mu_t/\mu_f < 1$. These definitions are the same as the forward locomotion behaviors except that the pose angle is defined to be in the opposite direction of the velocity direction, as opposed to aligned with it in the forward behavior cases. Though not considered, for these backward trajectories, the value of μ_b might be expected to have a larger effect on the trajectory behaviors as compared to forward and sidewinding trajectories.

Supplementary Note 3 — Steering rate, pose angle, and effective speed maps

In this note we provide additional field maps of the steering rate $\dot{\theta}$, pose angle γ, and effective speed $|\mathbf{v}_{\text{eff}}|$ to illustrate how these quantities vary for different friction ratios μ_t/μ_f, Froude numbers Fr, and lateral to horizontal wavenumber ratio λ.

Supplementary Note 3.1 — Impact of transverse friction ratio

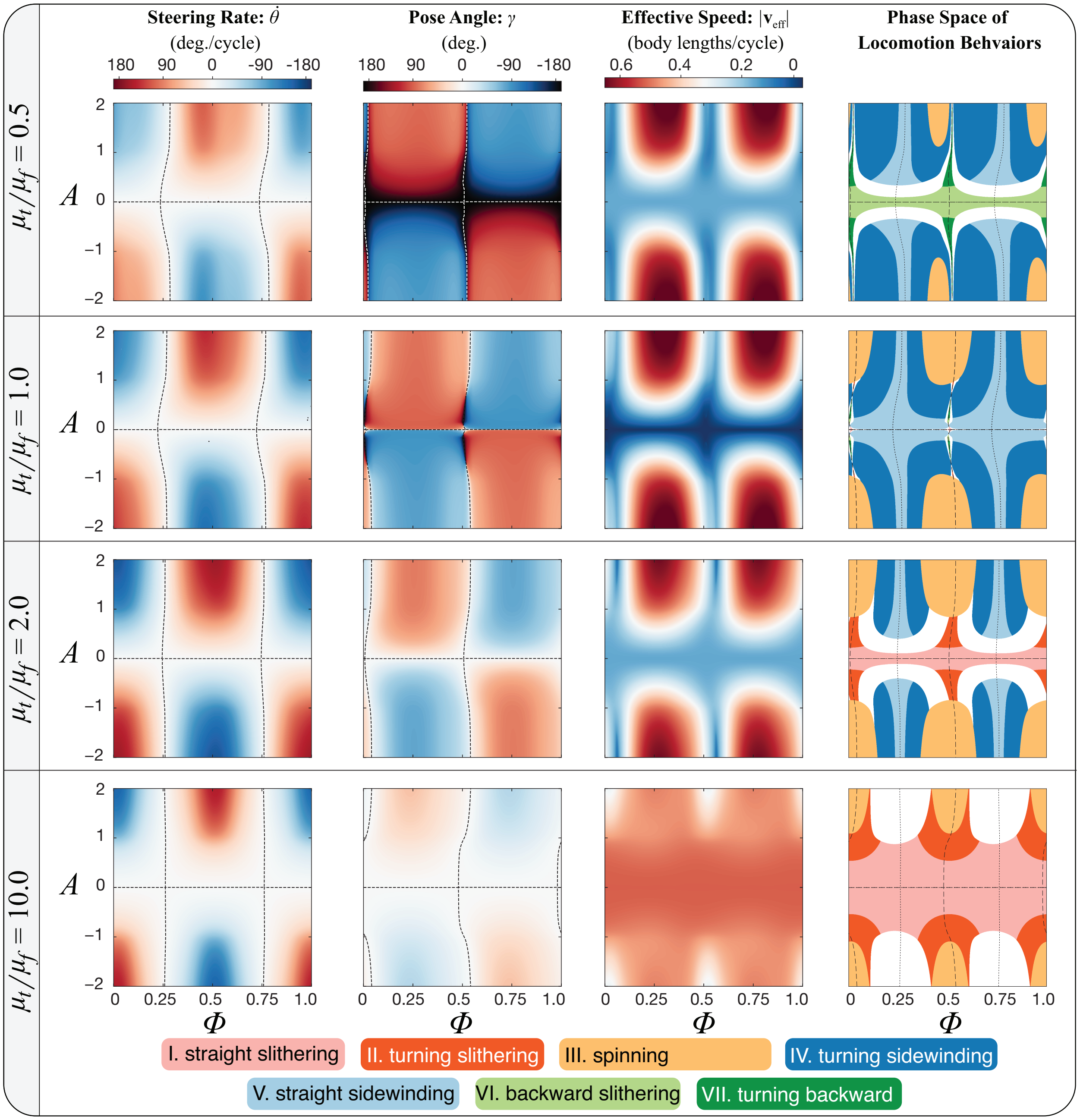

Supplementary Figure 3. Field maps of the steering rate $\dot{\theta}$, pose angle γ, and effective speed $|\mathbf{v}_{\text{eff}}|$ for the four different friction ratios μ_t/μ_f considered in the main text. Field maps are plotted with the same color range across friction ratios. As μ_t/μ_f increases, the pose angle tends towards $\gamma = 0$ for all A and Φ. Similarly, the effective speed $|\mathbf{v}_{\text{eff}}|$ increases and becomes progressively uniform for higher friction ratios.

Supplementary Note 3.2 — Impact of Froude number

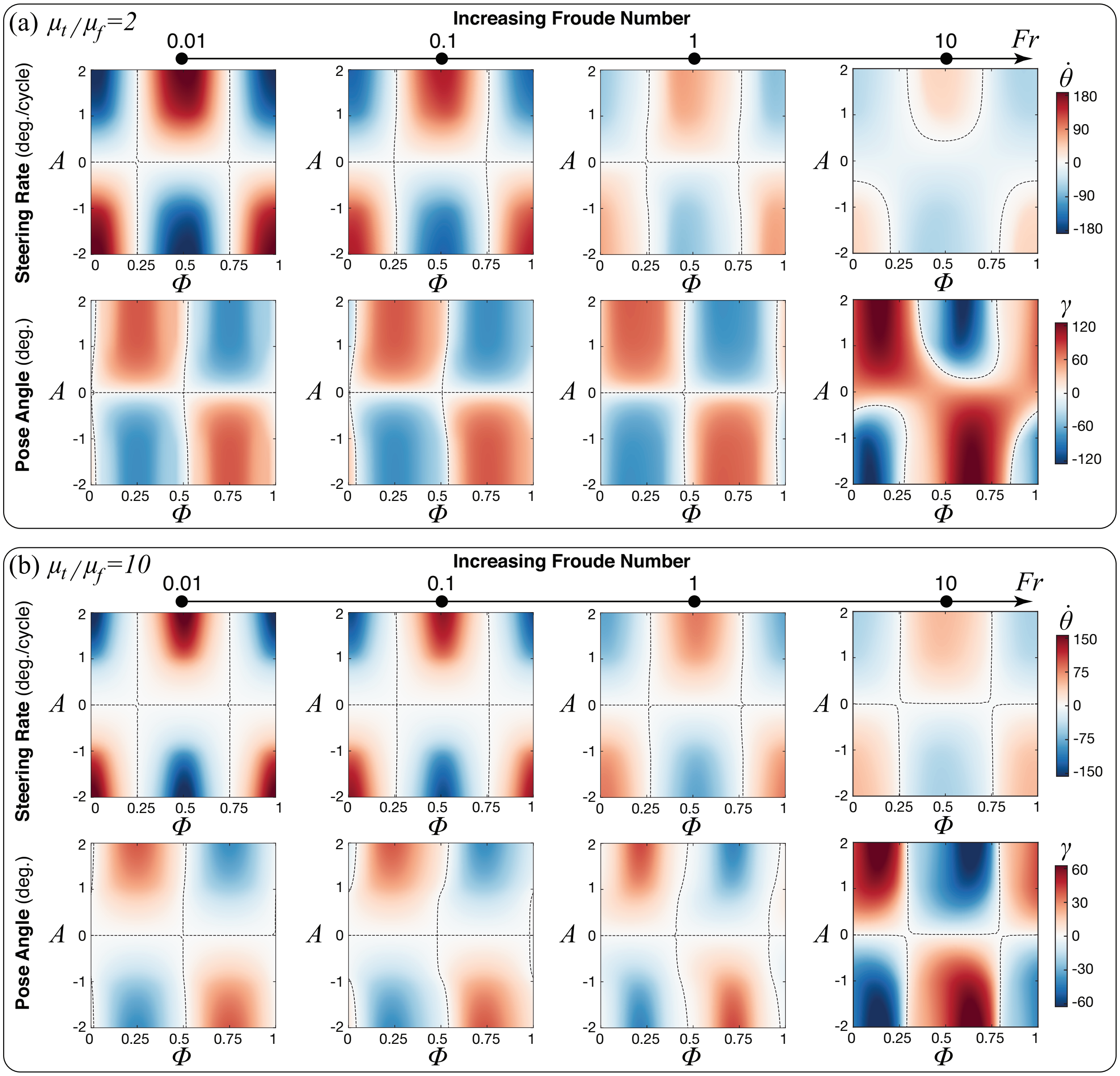

Supplementary Figure 4. Steering rate $\dot{\theta}$ and pose angle γ over a range of Froude numbers for (a) $\mu_t/\mu_f = 2$ and (b) $\mu_t/\mu_f = 10$. For $Fr \in [0, 01, 1.0]$ the steering rate and pose angle are broadly equivalent, justifying our analysis over a single Froude number ($Fr = 0.1$). As the Froude number exceeds values found in both biological and robotic snakes ($Fr = 10$), the behavior of the pose angle begins to exhibit substantially different trends. Dotted lines are zero contours.

Supplementary Note 3.3 — Impact of lateral to horizontal wavenumber ratio

The lateral, planar curvature of the snake is prescribed as $\kappa(s,t) = \epsilon \cos(2\pi k(s+t))$. For all cases considered in the main text, $\epsilon = 7.0$ and $k = 1$. The horizontal body lifting wave is modeled as friction modulation along the length of the snake through use of the non-dimensional scaling factor $\hat{N}(s,t) = \max\{0, A\cos(2\pi k_l(s+t+\phi)) + 1\}$. We define the ratio of these two lateral and lifting waves as $\lambda = k_l/k$. Two of the three stereotypical gaits considered in the main text are defined in terms of this ratio: asymmetric

($\lambda = 1$) and symmetric ($\lambda = 2$) friction modulation, which represent the cases of out-of-plane body lift by the snake. As mentioned in the main text, at steady-state motion, integrating over the body length and undulation period yields zero net force $\mathbf{F}_{net} = \int_0^1 \int_0^1 \mathbf{F}(s,t)\, ds\, dt$ and torque $\mathbf{T}_{net} = \int_0^1 \int_0^1 (\mathbf{x} - \bar{\mathbf{x}}) \times \mathbf{F}(s,t)\, ds\, dt$ for both symmetric body lifting and planar gait cases. In the symmetric body lifting case this means that the snake travels in a straight trajectory for all A and Φ, as opposed to the turning and sidewinding behaviors demonstrated by the asymmetric lifting case.

Supplementary Fig. 5 demonstrates how the the symmetric body lifting case exhibits only straight forward locomotion behavior over a range of phase offsets Φ and friction ratios μ_t/μ_f (for a fixed $A = 1$). The range of different steering and pose behaviors exhibited by the asymmetric lifting case instead is evident in comparison. For both asymmetric and symmetric body lifting, the effective velocity of the snake varies for both μ_t/μ_f and Φ. Maximum and minimum effective velocities are found at $\mu_t/\mu_f = 1$ for asymmetric and symmetric lifting, respectively. At low to moderate friction ratios there is a strong dependence on Φ. As μ_t/μ_f increases, the effective velocity behavior begins to lose its dependence on Φ and both lifting behaviors exhibit similar effective velocities.

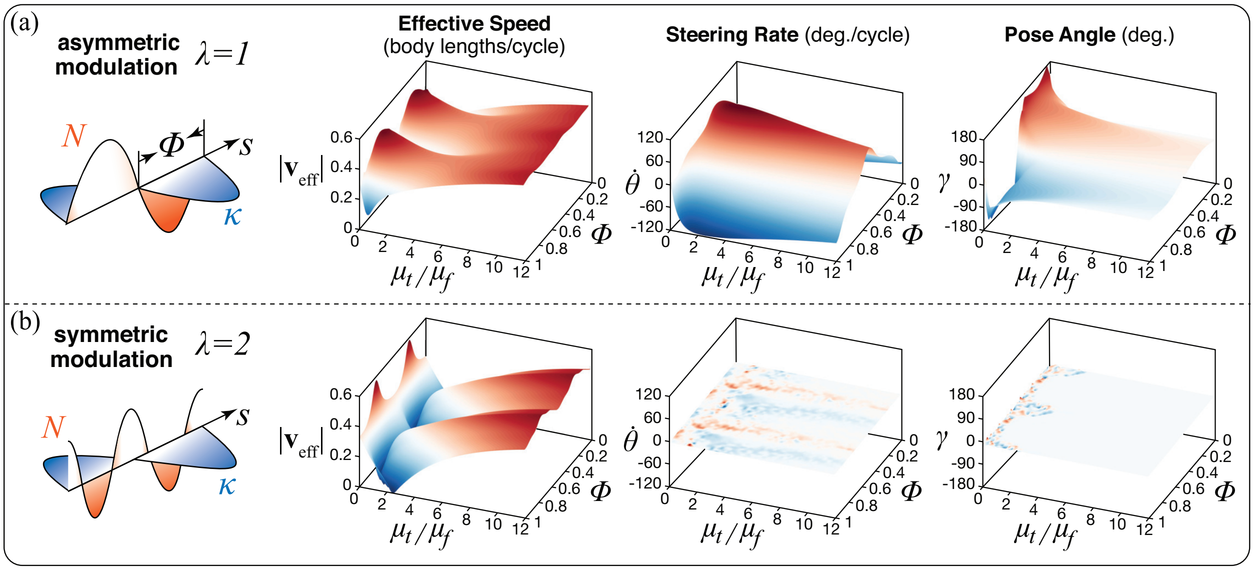

Supplementary Figure 5. Trajectory metrics for (a) asymmetric friction modulation and (b) symmetric friction modulation over different friction ratios μ_t/μ_f and phase offset between lateral and horizontal waves Φ. All cases are for a constant lifting amplitude $A = 1$. For all μ_t/μ_f and Φ, symmetric modulation results in $\dot{\theta} = 0$ and $\gamma = 0$, so that the snake travels in a forward direction with no turning behavior, while asymmetric modulation yields a variety of different steering and pose behaviors.

Supplementary Note 3.4 — Impact of modulation functional form

In the main text, we consider a cosine form of the modulating wave $\hat{N}$, chosen for consistency with the lateral wave form κ. Here, we test the snake model with two alternative modulation functions to show that the particular form of $\hat{N}$ does not significantly impact the locomotory behavior of the snake, as long as temporal decoupling with κ is achieved. As presented in Supplementary Fig.6, we use the same numerical protocol to generate the filed maps of snakes' steering rate and pose angle under exponential and linear modulation waves. Both scenarios yield quantitatively similar results relative to the cosine wave of the main text. Note that we still apply the normalization $N = \hat{N}/\int_0^1 \hat{N} ds$.

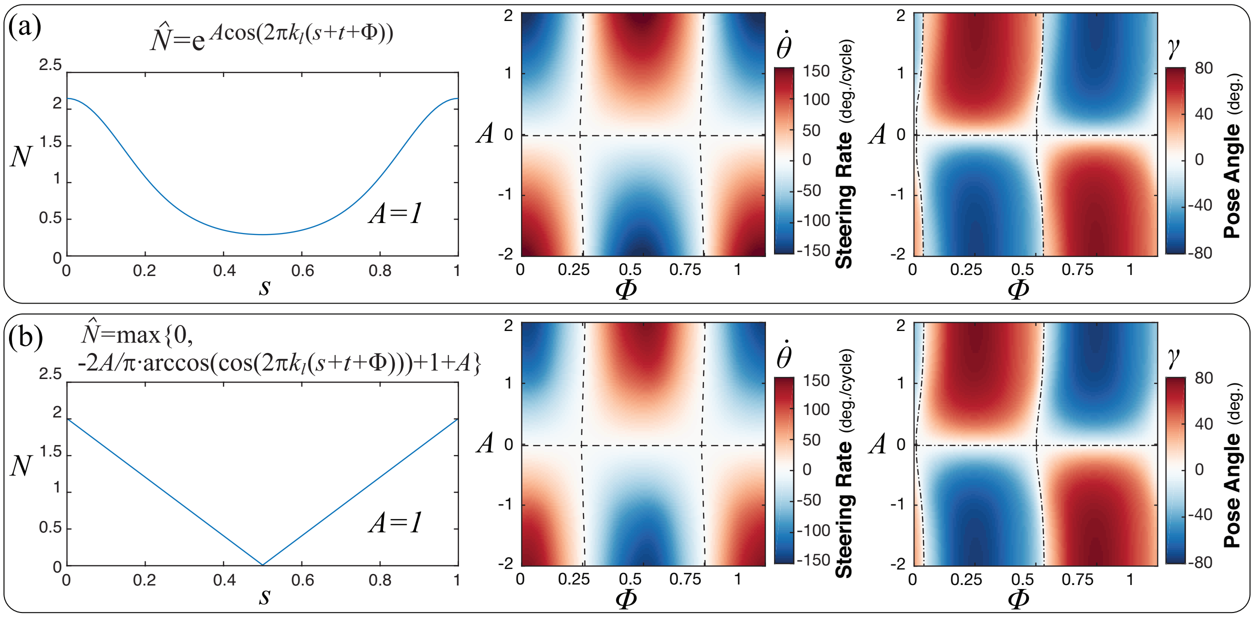

Supplementary Figure 6. Field maps of the steering rate $\dot{\theta}$ and pose angle γ under (a) exponential and (b) linear modulation waves.

Supplementary Note 4 — Simulating snakes using Cosserat rods

Supplementary Note 4.1 — Muscular activations and surrounding physics

As introduced in the main text, we utilize a 3D simulation approach to validate our planar analytical model and to study the interfacial dynamics of the snake with heterogeneous frictional terrains. We model the snake using an elastic filament described by Cosserat rod theory (Supplementary Fig. 7a), which has the governing equation stated in the Method section. Here, we provide detailed information about our numerical approach to account for the environmental effects and internal muscular activities of the snake.

Following the approach introduced in (*1*), we model the ground as a soft boundary to allow snake's interpenetration due to gravity, which will be eventually balanced out by the ground response force $\mathbf{F}_g$, written as

$$\mathbf{F}_g(s) = H(\epsilon) \cdot (F_{ng}(s) + k_g\epsilon - \gamma_g \mathbf{v}(s) \cdot \mathbf{n}_g)\, \mathbf{n}_g \tag{6}$$

where $\mathbf{n}_g$ is the normal direction of the ground, F_{ng} is the normal component of the overall forces experienced by the snake, $\mathbf{v}(s)$ is the local velocity of the snake and ϵ is the local interpenetration, k_g and γ_g are coefficients introduced to account for the elasticity and dissipation of the ground, respectively, and $H(\epsilon)$ is the Heaviside function that enables this response force only at the snake parts that are in contact with the ground ($\epsilon \geq 0$), which is assumed to be the $z = 0$ plane in all simulations. The friction model is based on a kinetic Coulomb friction model that incorporates the scaling factor p to account for ground heterogeneities. Therefore, the frictional force can be written as $\mathbf{F}_\mathrm{f} = -\mu_f p F_{ng} \cdot \boldsymbol{\mu}(s,t)$, where $\boldsymbol{\mu}(s,t)$ captures anisotropic friction effects and has the same expression as Eq. 5 in the Method section.

The filament in simulation is activated by muscular torques in both lateral and vertical directions, which produce the lateral undulation and lifting body wave of the snake, respectively. Muscular activations in both directions are modeled as traveling wave propagating along the filament from head to tail, and have the magnitude T_m expressed as

$$T_m = \beta_m(s) \cdot \sin\left(\frac{2\pi}{\tau_m}t - 2\pi\Phi - 2\pi s\right), \tag{7}$$

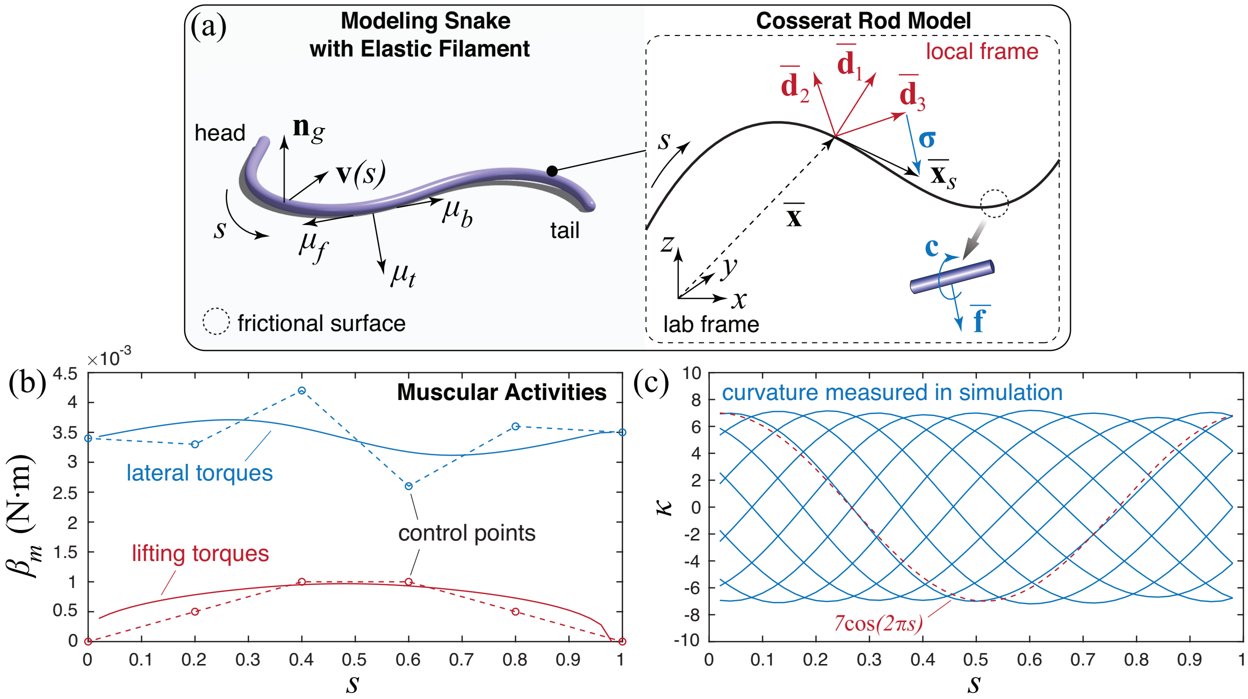

Supplementary Figure 7. (a) Using Cosserat rod theory to model the snake as an elastic filament. (b) Muscular activation implemented in simulation (solid curves) in both lateral and vertical directions, generated using cubic B-spline functions defined by six control points (dashed curves). (c) Comparing the non-dimensionalized lateral curvature of the simulated snake during movement against the stereotypical curvature function κ.

where τ_m is the undulation period fixed to be 2 seconds in all simulations, Φ indicates the phase offset of the lifting muscular signal, which is set to be 0 in the lateral torques, $\beta_m(s)$ denotes the cubic B-spline function that represents the amplitude of the torque and is characterized by 6 equally spaced control points along the filament. The selected control points together with the resulting $\beta_m(s)$s are plotted in Supplementary Fig. 7b for both lateral and lifting cases. As shown in Supplementary Fig. 7c, the snake actuated by the given lateral torques recovers the prescribed lateral curvature employed in our planar model (see Methods).

Supplementary Note 4.2 — Extended simulation methods and results

As mentioned in the main text, snakes simulated in Fig.4 employ the same lateral muscular activities, thus their diverse behaviors stem solely from the effect of friction modulation. Here, we provide detailed information about our simulation setups and environmental settings to reproduce the results of each test case.

In **Fig. 4b**, snakes employ lifting muscle torques of different magnitude and phase offset. The magnitude is captured by AT_m^{max}, where $A \in [0, 1]$ denotes the lifting ratio and T_m^{max} is the maximum torque allowed in the simulation, expressed as Eq. 7, with $\beta_m(s)$ values given in Supplementary Fig. 7b. The phase spaces are constructed by the result of 1681 snake simulations (on a 41×41 equally spaced grid) with friction ratio $\mu_t/\mu_f = 2$. Similarly, the steering and pose of snakes having $\mu_t/\mu_f = 10$ are explored by deploying the same simulation protocol, generating the phase spaces presented in Supplementary Fig. 8a. The result demonstrates the decreased steering and rotational capabilities of the snake at high friction ratios, with corresponding phase spaces exhibiting color patterns consistent with Fig. 4b. This result complements our study at $\mu_t/\mu_f = 2$, providing validations against the planar model across frictional environments.

Supplementary Fig. 8b plots snake trajectories for friction condition $\mu_t/\mu_f = 2$ in addition to the result in **Fig. 4c**, with $A = 1$ and extended range of $\Phi \in [0, 1]$. Trajectories of snakes having $\mu_t/\mu_f = 10$ are

plotted in Supplementary Fig. 8c, for (1) $\Phi = 0$ and $A \in [0, 1]$ and (2) $A = 1$ and $\Phi \in [0, 1]$.

Moving to the studies of heterogeneous terrains, we demonstrate the results in **Fig.4e-j** by employing fast moving snakes having friction ratio $\mu_t/\mu_f = 10$.

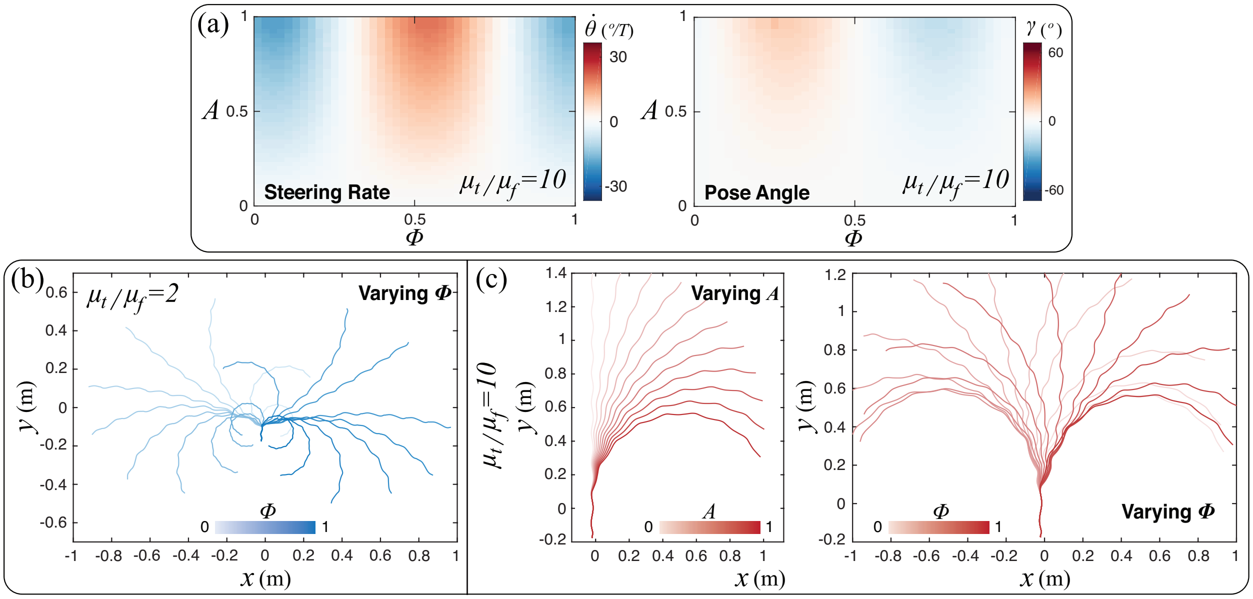

Supplementary Figure 8. (a) Phase spaces of snakes' steering and pose angle under $\mu_t/\mu_f = 10$, plotted with the same color ranges as in $\mu_t/\mu_f = 2$ case. (b) Trajectories of snakes having $\mu_t/\mu_f = 2$ with different phase offsets. (c) Trajectories of snakes having $\mu_t/\mu_f = 10$ with varying amplitude or phase offsets.

In **Fig. 4e**, we show how the snake's diffraction behavior observed in experiment (2) can be recovered by friction modulation. In our simulation, high frictional patches have a scaling factor $p = 30$, meaning that friction coefficients in all directions are scaled up 30 times. To reproduce the experimental setup, patches are designed to have radius $r_p = 7.2$ mm (combining post radius 3.2 mm and snake radius 4 mm in (2)) and center-to-center distance $d_p = 23$ mm. The initial position of the snake's head can vary within the dashed box in the figure, so that snakes with different starting point will interact with the patches at different phases during undulation. The box has width 0.14 m (snakes further away will not interact with patches) and length 0.178 m (distance traveled by the snake in one cycle). We simulated 525 snakes with uniformly distributed head positions to generate the probability density function of the main text. Half of the trajectories are included in the trajectory plot, to avoid excessively occluding the figure.

In **Fig. 4g**, snakes traveling through a horizontal frictionless strip (where $p = 0$) are refracted/reflected in different directions due to friction modulation at the interface. The strip is set to be infinitely long in the x direction and centered at $y = 0.2$. All snakes start out with their heads being positioned at the origin (38 simulations).

Fig. 4h presents three examples of passive control of snake trajectory using frictionless surface patterns. (1) Two parallel strips are centered at $y = -0.3225$ and $y = 0.7036825$, and three snakes move towards the strips with initial bearings $\alpha = 30°$ (blue), $-30°$ (red) and $-10°$ (green), respectively. (2) Strips that are perpendicular to the snake's moving direction can be designed to rather precisely modulate the deflection of the snake's trajectory. Snakes that encounter the strip centered at $y = 0.2$ are deflected to the left (blue) while snakes that encounter the strip centered at $y = 0.28$ are deflected to the right (red). Here, we note that when the snake employs the same lateral wave, the distance between the strip and origin of the snake d can be though of as the phase offset Φ for 3D gaits, since they both determine the onset of friction modulation in one snake cycle. We demonstrate that when we offset the strip location by 0.8, which is close to half of

the snake's displacement in one cycle (0.178m), the snake trajectories are reflected. This is reminiscent of a scenario in which two 3D snakes have $\Delta\Phi = 0.5$. In this case, indeed, the two trajectories will also be symmetric (as shown in Supplementary Fig. 8b,c). (3) Six strips are radially arranged to guide the turning motion of snakes. The radial pattern is centered at (-0.83044147,0.2), with the angle between every two strips being $34.21°$. The starting points for the three snakes are (0.2,0), (0,0), and (-0.2,0) respectively.

In **Fig. 4i**, the circular friction patterns are modeled using radial basis functions $p(d_c) = 1+p_{max}e^{-(\sigma d_c)^2}$, where d_c is the distance between any point on the snake and the pattern center, p_{max} (≥ -1) is the elevation/depth at the pattern center, and σ controls the size of the pattern. We use $\sigma = 3$ for larger patterns and $\sigma = 5$ for smaller patterns in the figure.

The frictional landscape in **Fig. 4j** is constructed using a series of radial basis functions expressed as

$$p = 1 + \sum_{i=1}^{N_p} p^i_{max} e^{-(\sigma^i d^i_c)^2} \tag{8}$$

where parameters for each radial function have the same definitions as in the previous case, with $N_p = 30$ being the total number of the radial functions. Values used to create the landscape are in the following list

$$\begin{bmatrix} x^i \\ y^i \\ \sigma^i \\ p^i_{max} \end{bmatrix} = \begin{bmatrix} 1 & 0.5 & 1 & 2 & 2.25 & 3.5 & 4 & 4.5 & 5 & 3.1 \\ 0.5 & -0.7 & -0.7 & 0.25 & -0.25 & -0.75 & -1 & -1 & -0.5 & 1.1 \\ 2.5 & 2.5 & 2.5 & 2.5 & 3 & 3 & 2.5 & 2.5 & 3 & 6 \\ 7 & 4 & 5 & 6 & 5 & -1 & -1 & -1 & -1 & -1/3 \end{matrix}$$

$$\begin{matrix} 3.2 & 3.3 & 1.3 & 1.6 & 1.9 & 2.15 & 2.4 & 5.5 & 5.8 & 6.5 \\ 0.75 & 0.3 & 2.3 & 2.25 & 2.1 & 1.95 & 1.8 & 0.55 & 0.9 & 0.7 \\ 3.5 & 4 & 6.5 & 5.5 & 5 & 6 & 7.5 & 3.5 & 4 & 3.5 \\ -1 & -1 & -0.5 & -1 & -1 & -1 & -0.5 & 7 & 7 & 3 \end{matrix}$$

$$\begin{matrix} 6.9 & 6.5 & 7.2 & 5 & 5.3 & 5.6 & 6.25 & 6.5 & 7 & 6.8 \\ 0.5 & -0.3 & -0.3 & 1.25 & 1.5 & 1.6 & 1.75 & 1.75 & 0.85 & 0.9 \\ 3.5 & 4.5 & 4 & 3 & 3 & 3 & 6 & 6 & 4.5 & 5 \\ 5 & 2 & 1 & -1 & -1 & -0.5 & -1 & -0.5 & -1 & -0.5 \end{bmatrix},$$

where (x^i, y^i) is the center of each radial function. p is then ensured to be non-negative by taking $\max\{0, p\}$.

Supplementary Note 4.3 — Simulation Parameters

Parameters relative to our numerical scheme are listed in the following table.

Supplementary Table S1. Simulation parameters

**Parameters**	**Values**	**Parameters**	**Values**
Time step	8×10^{-6}s	Simulation time	> 20s
Number of elements in snake	50	Snake density	1g/cm^3
Snake Young's modulus	1MPa	Poisson Ratio	0.5
Ground stiffness k_g	1kg/s^2	Ground damping γ_g	10^{-6}kg/s

Supplementary References

1. Gazzola, M., Dudte, L. H., McCormick, A. G. & Mahadevan, L. Forward and inverse problems in the mechanics of soft filaments. *Royal Society Open Science* **5** (2018).

2. Schiebel, P. E. *et al.* Mechanical diffraction reveals the role of passive dynamics in a slithering snake. *PNAS* **116**, 4798–4803 (2019).
