# Supplementary material for: Friction modulation in limbless, three-dimensional gaits and heterogeneous terrains
Source: Nat Commun. 2021 Oct 19;12:6076. doi: 10.1038/s41467-021-26276-x (PMC8526626; doi:10.1038/s41467-021-26276-x)
Supplement: Supplementary file 3 — Description of Additional Supplementary Files [file 41467_2021_26276_MOESM3_ESM.pdf]

<u>Description for additional Supplementary Material</u>

Supplementary Movie 1: Classifications of locomotion behaviors. Results are obtained using the planar snake model.

Supplementary Movie 2 : A demonstration of sidewinding locomotion through 3D elastic simulations, where lifting torque waves enable out-of-plane body deformations.

Supplementary Movie 3 : Controlling snake locomotion through lifting amplitude and phase offset (5x speed).

Supplementary Movie 4 : Channeling a snake through two parallel frictionless strips (5x speed).

Supplementary Movie 5 : Controlling a snake’s turning trajectory through radially patterned frictionless strips (5x speed).

Supplementary Movie 6 : Snakes with different starting locations passively meander through an heterogeneous frictional contour map (5x speed).
